# Bioinformatics Analysis of Lactylation-related Biomarkers and Potential Pathogenesis Mechanisms in Age-related Macular Degeneration

**DOI:** 10.2174/0113892029291661241114055924

**Published:** 2025-01-02

**Authors:** Chenwei Gui, Yan Gao, Rong Zhang, Guohong Zhou

**Affiliations:** 1 The First Clinical Medical College, Shanxi Medical University, Taiyuan, Shanxi, China;; 2 Department of Ophthalmology, Shanxi Eye Hospital Affiliated to Shanxi Medical University, Taiyuan, Shanxi, China

**Keywords:** Age-related macular degeneration, bioinformatics, machine learning, hub genes, lactylation, immune infiltration

## Abstract

**Background:**

Lactylation is increasingly recognized to play a crucial role in human health and diseases. However, its involvement in age-related macular degeneration (AMD) remains largely unclear.

**Objectives:**

The aim of this study was to identify and characterize the pivotal lactylation-related genes and explore their underlying mechanism in AMD.

**Methods:**

Gene expression profiles of AMD patients and control individuals were obtained and integrated from the GSE29801 and GSE50195 datasets. Differentially expressed genes (DEGs) were screened and intersected with lactylation-related genes for lactylation-related DEGs. Machine learning algorithms were used to identify hub genes associated with AMD. Subsequently, the selected hub genes were subject to correlation analysis, and reverse transcription quantitative real-time PCR (RT-qPCR) was used to detect the expression of hub genes in AMD patients and healthy control individuals.

**Results:**

A total of 68 lactylation-related DEGs in AMD were identified, and seven genes, including *HMGN2*,* TOP2B*, *HNRNPH1*, *SF3A1*, *SRRM2*, *HIST1H1C*, and *HIST1H2BD* were selected as key genes. RT-qPCR analysis validated that all 7 key genes were down-regulated in AMD patients.

**Conclusion:**

We identified seven lactylation-related key genes potentially associated with the progression of AMD, which might deepen our understanding of the underlying mechanisms involved in AMD and provide clues for the targeted therapy.

## 1 INTRODUCTION

Age-related macular Degeneration (AMD) is a prevalent and progressive ocular ailment that predominantly impacts the elderly. AMD can lead to profound visual impairment or complete loss of vision in older individuals and has become a substantial public health concern [1, 2]. The macula, responsible for central vision and intricate visual activities like reading, driving, and facial recognition, is primarily affected by AMD [3-5]. The etiology of AMD remains intricate and multifaceted, involving a confluence of genetic predisposition, environmental influences, and lifestyle choices [6-8]. In recent times, the progress made in high-throughput sequencing technologies and bioinformatics tools has boosted a comprehensive investigation of the molecular underpinnings of AMD. The utilization of transcriptomics has yielded significant knowledge regarding the biological pathways implicated in the progression of this disease. Moreover, the implementation of computational methodologies, such as machine learning algorithms and network analyses, has played a significant role in promoting the recognition of new biomarkers and potential therapeutic targets for AMD [9, 10].

The process of lactylation modification, or lactylation, refers to the chemical procedure of incorporating lactyl groups into biomolecules [11-13]. This modification has garnered considerable attention within the biomedical research community due to its potential utility in diverse therapeutic and diagnostic approaches. Nevertheless, the precise involvement of lactylation in AMD remains uncertain.

In this investigation, a gene expression profile was obtained from the Gene Expression Omnibus (GEO) database to discern differentially expressed genes (DEGs) between AMD and control samples. The function of these DEGs was further investigated using bioinformatics analysis. Specifically, DEGs related to lactylation were selected and analyzed using three machine-learning approaches: Random Forest, Support Vector Machines (SVM), and Least Absolute Shrinkage and Selection Operator (Lasso). From these analyses, seven hub genes were ascertained and subsequently subjected to immune cell infiltration analysis and unsupervised clustering.

## 2 MATERIALS AND METHODS

### 2.1 Data Collection

Two AMD datasets (GSE29801, GSE50195) were collected from the GEO database at https://www.ncbi.nlm.nih.gov/geo/query/acc.cgi?acc=GSE29801 and https://www. ncbi.nlm.nih.gov/geo/query/acc.cgi?acc=GSE50195, respectively. Both profiles were derived from human samples. GSE29801 included 79 RPEChoroid samples from AMD patients and 96 control samples. For GSE50195, 9 RPEChoroid samples from AMD patients and 7 from control individuals were included. The lactylation-related genes utilized in this study were sourced from previously published research [14-16].

### 2.2 Identification of Lactylation-related DEGs

After merging the expression data from GSE29801 and GSE50195, the batch effect was eliminated with the “sva” package in R software. DEGs were then screened utilizing the “limma” package in order to ascertain DEGs between AMD patients and controls based on the merged expression profile. A significance threshold of *P*-value < 0.05 was applied. The lactylation-related DEGs in AMD were screened out by intersecting the DEGs in AMD and lactylation-related genes obtained from previous studies.

### 2.3 Enrichment Analysis

To gain insight into the biological significance and functions of the identified DEGs, Gene Ontology (GO), Kyoto Encyclopedia of Genes and Genomes (KEGG) enrichment analysis, and Gene Set Enrichment Analysis (GSEA) were conducted with the “clusterProfiler” package.

### 2.4 Hub Gene Selection

In order to ascertain hub genes linked to AMD, LASSO, random forest, and SVM-RFE algorithms were employed [17]. LASSO as a dimension is superior in the evaluation of high-dimensional data compared with the regression analysis reduction method. LASSO was performed using the R package “glmnet” [18]. Random Forest analysis uses a supervised machine learning approach, recursive feature elimination (RFE), for ranking the importance of lactylation-related DEGs using the R package “randomForest” [19]. The top ten genes ranked by importance were selected as characteristic genes. SVM-RFE is a small-sample learning method that ranks features based on recursion and has good performance in relevant characteristic selection. The feature genes were screened when the classifier had the minimum error. SVM-RFE was performed using the R package “kernlab” [20]. Then, a Venn diagram was applied to visualize the hub genes common to the three algorithms.

### 2.5 Tumor Microenvironment Analysis

The abundance of immune cells in patient samples was analyzed by “ssGSEA” algorithm using “GSVA” R package, followed by computing the correlation between levels of key genes and immune cell abundance and visualization using “ggplot2” R package.

### 2.6 Gene Regulatory Network

Transcriptional and post-transcriptional gene regulation are critically involved in diverse biological processes and cellular functions. The potential regulatory networks of key genes were predicted with the Regnetwork database (www.regnetworkweb.org). The networks were visualized relying on Cytoscape software.

### 2.7 Consensus Molecular Clustering Based on Key Genes

To group patients into distinct clusters, a consensus clustering algorithm was employed with the R package “ConsensusClusterPlus,” which relied on the expression characteristics of the seven hub genes.

### 2.8 Human Samples

The blood samples were obtained from AMD patients (n=20) and age-, gender-matched healthy control donors (n=20) without retinopathies in our hospital. The serum was collected after centrifuging at 400× g for 15 min, aliquoted and stored at −80 °C until use. The written informed consent was obtained from all participants. This study was conducted using open-source data and has no ethical issues or conflicts of interest.

### 2.9 Reverse Transcription Quantitative Real-time PCR (RT-qPCR)

TRIzol reagent (Invitrogen) was used for total RNA extraction. Then cDNAs were synthesized *via* reverse transcription using a PrimeScript RT Kit (Takara, Dalian, China). PCR was conducted using the StepOnePlus Real-Time PCR System (Thermo Fisher, USA). Expression levels of key genes were quantified with the 2^−∆∆Ct^ method and normalized to GAPDH. The primer sequences used were as follows: *TOP2B*-F:5’-TACGATGATGCAGAATCTCTG-3’, *TOP2B*-R:5’-GTGAGAACCATCTTGATCCTG-3’, *HMGN2*-F:5’-GAGAAGGTACCCAAAGGGA-3’, *HMGN2*-R:5’-CTTCAGCTTTCTGTGCCTG-3’; *SRRM2*-F:5’-TAAGCATAGGTCTCCCACTC-3’, *SRRM2*-R:5’-GCTGGTGTTGTACTTCGAG-3’; *HNRNPH1*-F:5’-GGGTATAACAGCATTGGCAG-3’, *HNRNPH1*-R:5’-ATCATAGCCTCCATAGCCTC-3’; *SF3A1*-F:5’-TTGGACTTCTTGACCCTCG-3’, *SF3A1*-R:5’-AATATCCAGACCTGGTGCG-3’; *HIST1H1C*-F:5’-GCCGTATCAAACTTGGTCTC-3’, *HIST1H1C*-R:5’-CCTTTCGTTTGCACCAGAG-3’; *HIST1H2BD*-F:5’-GTAACTTTGCCAAGGGAGAG-3’, *HIST1H2BD*-R:5’-CAGGCAGATGAGACTTCCT-3’; *GAPDH*-F:5’-TCAAGATCATCAGCAATGCC-3’, *GAPDH*-R: 5’-CGATACCAAAGTTGTCATGGA-3’.

## 3 RESULTS

### 3.1 Screening of DEGs between AMD and Control Samples

**Fig. (1):**
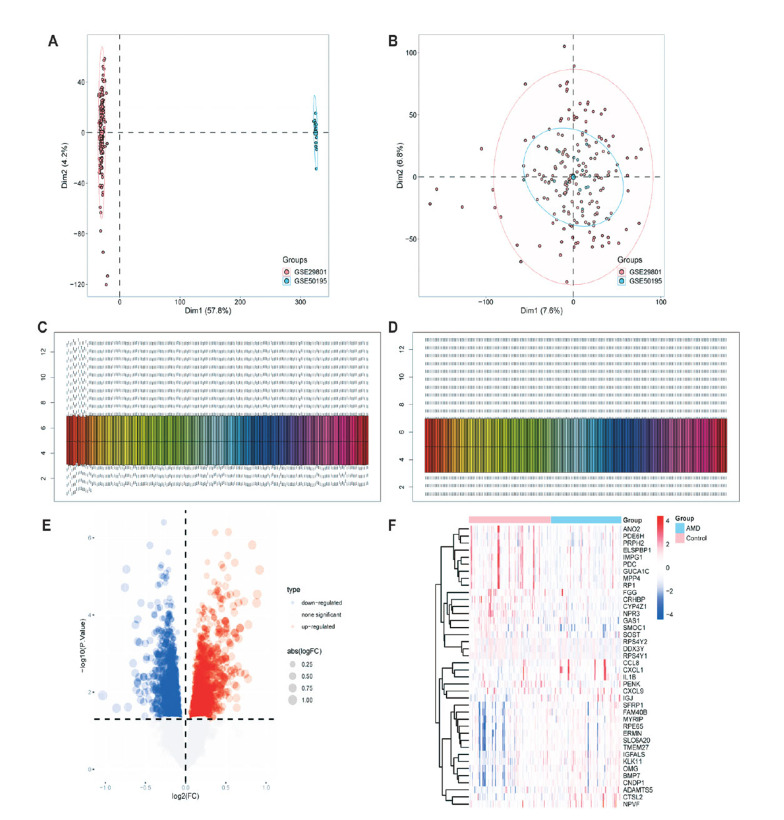
Data processing and selection of DEGs. (**A-B**) PCA plot of GSE50195 and GSE29801 before (**A**) and after (**B**) merging. Boxplots exhibited the batch effects of expression profiles (**C**) before and (**D**) after homogenization. (**E**) Volcano plots showed the gene expression pattern in the merged gene expression matrix. (**F**) Heatmap exhibited the top 20 differently expressed genes.

Two expression profiles in GSE29801 and GSE50195 were merged and normalized before further analysis (Fig. **1A**-**D**). The identification of DEGs was performed based on the merged data with a *P*-value <0.05 as the threshold value. The DEGs were shown in a volcano plot and heatmap (Fig. **1E** and **F**). Results showed that a total of 1273 genes exhibited up-regulation, while 1417 genes demonstrated down-regulation in the AMD group.

### 3.2 Enrichment Analyses of DEGs

**Fig. (2):**
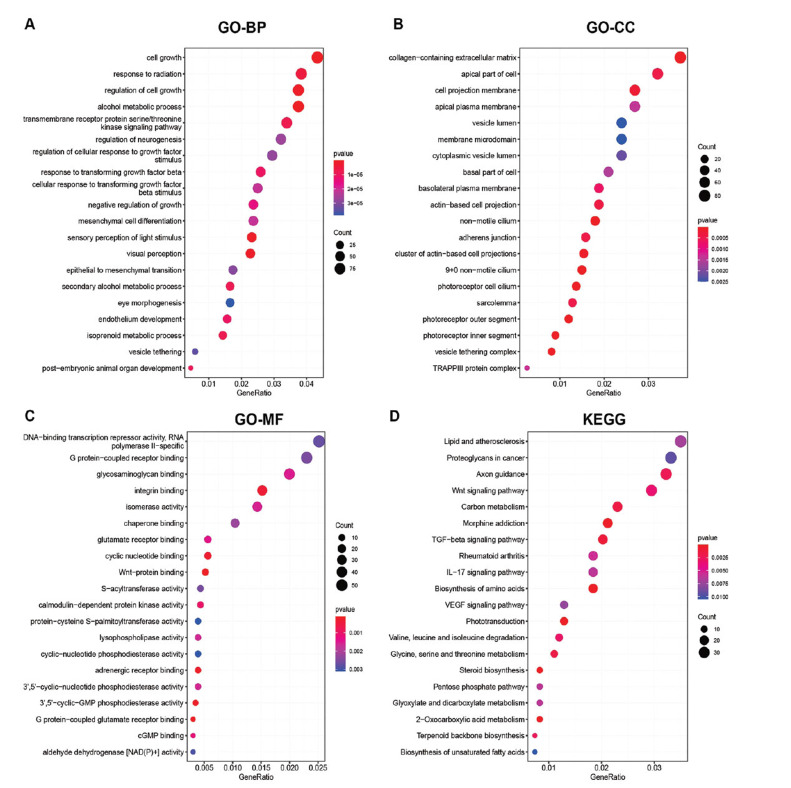
GO and KEGG analyses for DEGs. (**A-C**) DEGs were subject to GO enrichment analysis and the enriched terms in (**A**) biological process (BP), (**B**) cellular component (CC), and (**C**) molecular function (MF) were presented. (**D**) The KEGG enrichment results of DEGs.

Functional enrichment analyses were conducted, and the GO analysis demonstrated that the DEGs were predominantly linked to biological processes like cell growth, response to radiation, and alcohol metabolic processes (Fig. **2A**). In terms of cellular components, the DEGs were related to terms such as collagen-containing extracellular matrix (ECM), apical part of the cell, and cell projection membrane (Fig. **2B**). Furthermore, the molecular functions of the DEGs included DNA-binding transcription repressor activity, RNA polymerase II-specific, G protein-coupled receptor binding, and glycosaminoglycan binding (Fig. **2C**). In addition, the KEGG analysis indicated the enrichment of lipid and atherosclerosis pathways, Axon guidance, Wnt signaling pathway, Carbon metabolism, Morphine addiction, and TGF-beta signaling (Fig. **2D**).

### 3.3 Screening of Lactylation-related DEGs

**Fig. (3):**
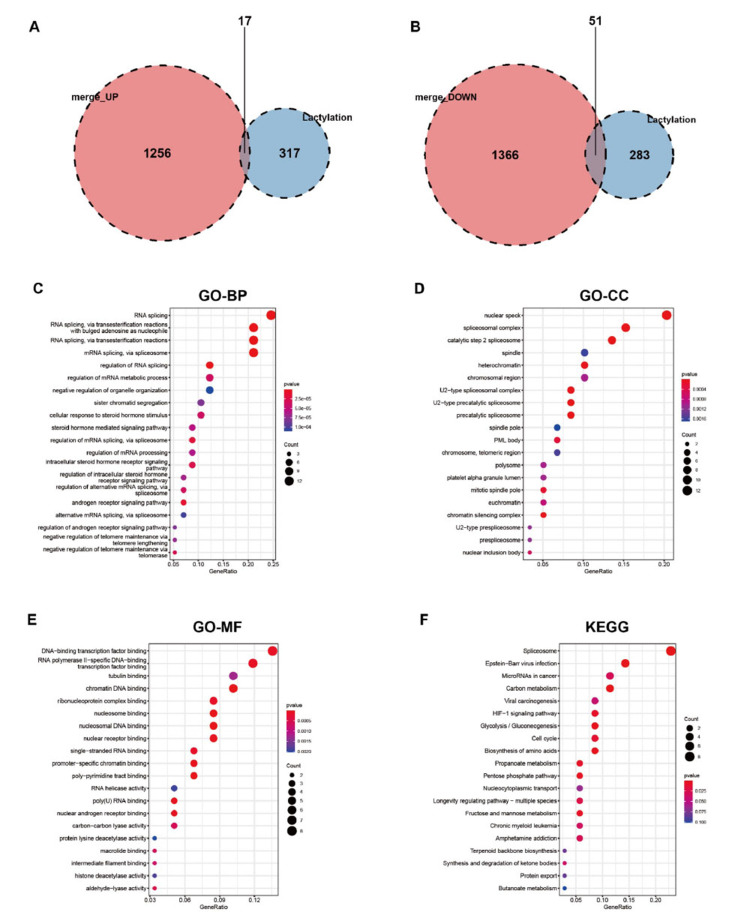
Screening of lactylation-associated DEGs. (**A-B**) A total of 17 lactylation-associated up-regulated genes and 51 down-regulated genes were presented in Venn diagrams. (**C-F**) GO and KEGG analyses of lactylation-associated DEGs.

**Fig. (4):**
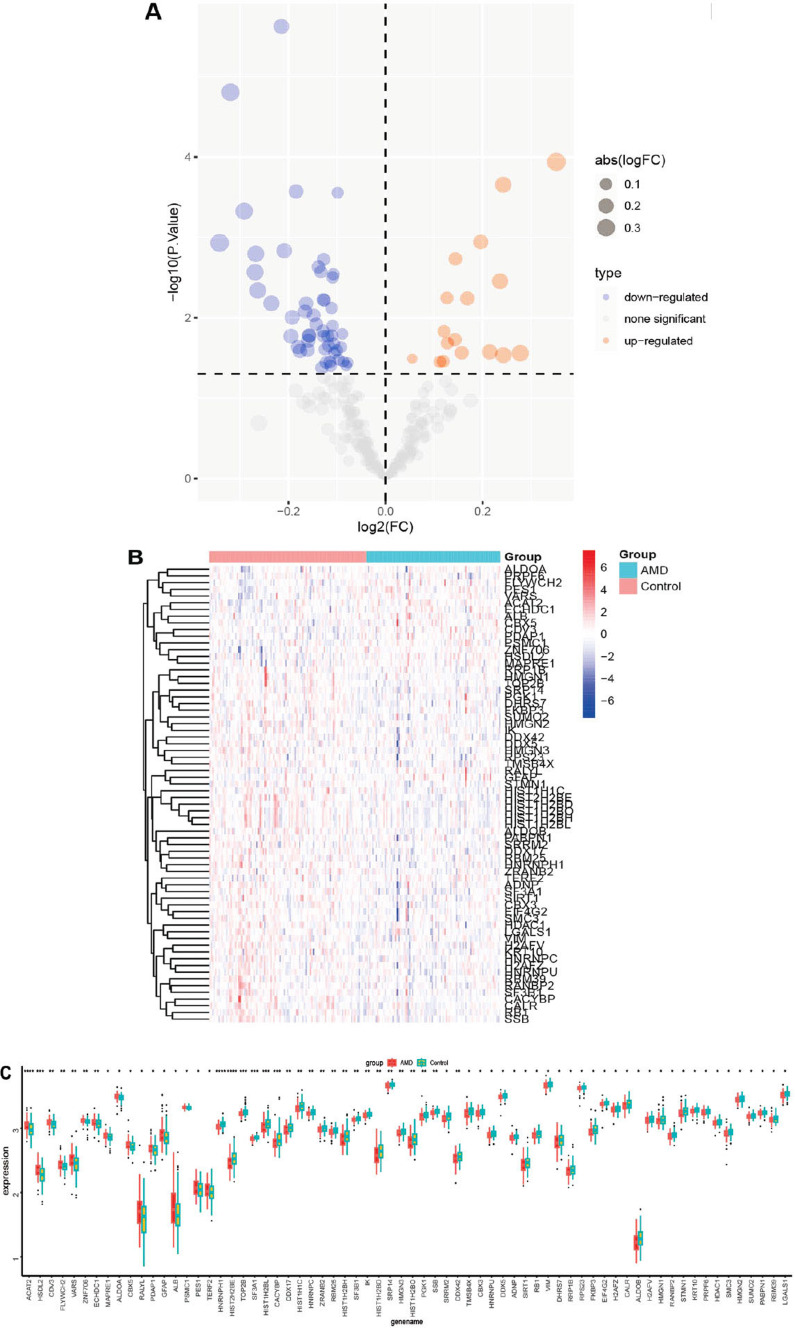
Expression pattern of lactylation-associated DEGs between AMD and control samples. (**A**) Volcano plot and (**B**) Heatmap exhibited the expression pattern of lactylation-associated DEGs. (**C**) Expression comparison of lactylation-associated DEGs between AMD and control samples.

Subsequently, the lactylation-related genes were selected from the pool of DEGs. Our findings revealed that out of the 68 lactylation-related DEGs, 17 genes exhibited up-regulation in AMD, while 51 genes displayed down-regulation (Fig. **3A** and **B**). Further analysis using GO demonstrated that the lactylation-related DEGs were predominantly linked to RNA splicing in biological processes (BP), nuclear speck in cellular components (CC), and DNA-binding transcription factor binding in molecular functions (MF) (Fig. **3C**). Additionally, the KEGG analysis demonstrated the enrichment of Spliceosome, Carbon metabolism, HIF-1 signaling pathway, and Glycolysis/Gluconeogenesis (Fig. **3D-F**). The 68 lactylation-related genes were additionally presented using a merged expression profiler. The volcanic diagram and heatmap of the 68 lactylation-related DEGs were depicted in Fig. (**4A**) and (Fig. **4B**), respectively. Fig. (**4C**) exhibited the differential expression of the 68 lactylation-related DEGs.

### 3.4 Screening of Key Genes Based on Machine Learning

**Fig. (5):**
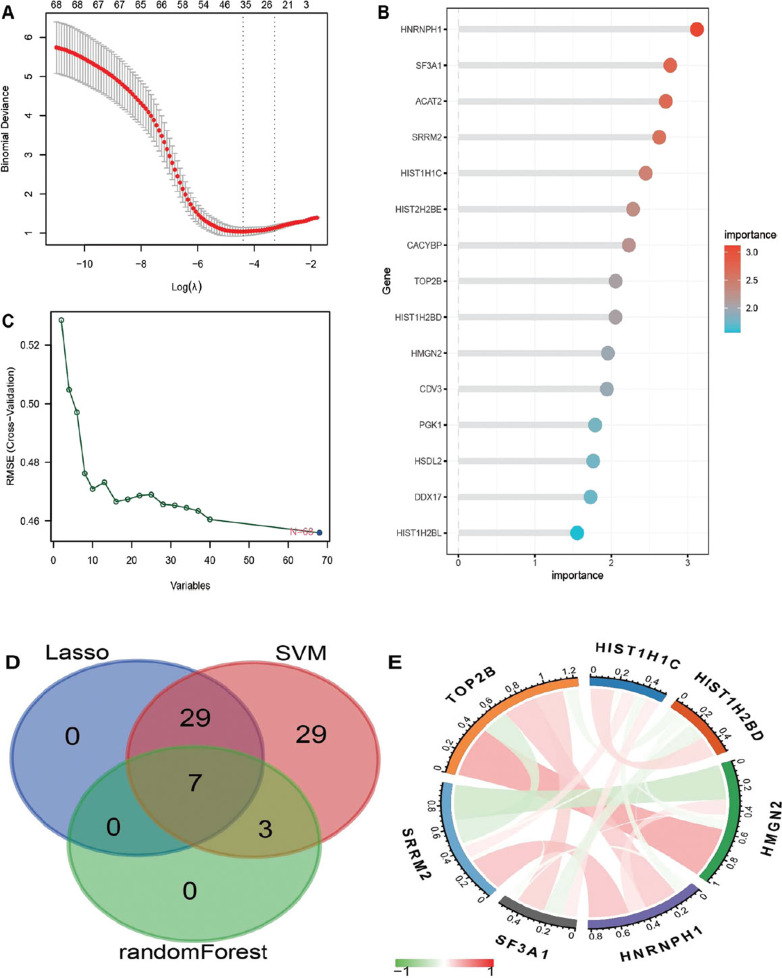
Hub gene selection. (**A**) LASSO algorithm, (**B**) random forest algorithm and (**C**) SVM-RFE algorithm were employed for selecting the feature genes. (**D**) A Venn diagram showed the common feature genes selected by three algorithms. (**E**) Expression correlation of selected hub genes was analyzed and visualized using a chord plot. The red lines indicate positive correlations, while the green lines signify negative correlations. The higher the color depth, the stronger the correlation.

We employed 3 machine-learning algorithms for hub gene selection from the acetylation-associated DEGs. Based on LASSO analysis, the minimum criteria were selected to construct the LASSO classifier based on superior accuracy in comparisons with 36 feature genes (Fig. **5A**). According to analysis using Random forest, feature genes were ranked by importance value, and the ten most important genes included *CACYBP, TOP2B*, *HNRNPH1*, *HIST1H2BD, HIST1H1C*, *HIST2H2BE, ACAT2, SF3A1*, *SRRM2*, and *HMGN2* (Fig. **5B**). Meanwhile, the SVM-RFE algorithm was used to identify the best feature gene combination, and the classifier was found to have the minimum error (0.458) when the feature count reached 68 (Fig. **5C**). Through the intersection of the results from three algorithms, a total of 7 characteristic genes (*HMGN2*, *SRRM2*, *TOP2B*, *HIST1H1C*, *SF3A1*, *HIST1H2BD,* and *HNRNPH1*; Fig. **5D**) were identified. Furthermore, the expression levels of these 7 key genes exhibited a significant positive correlation with each other (Fig. **5E**).

### 3.5 Analysis of Immune Cell Distribution

**Fig. (6):**
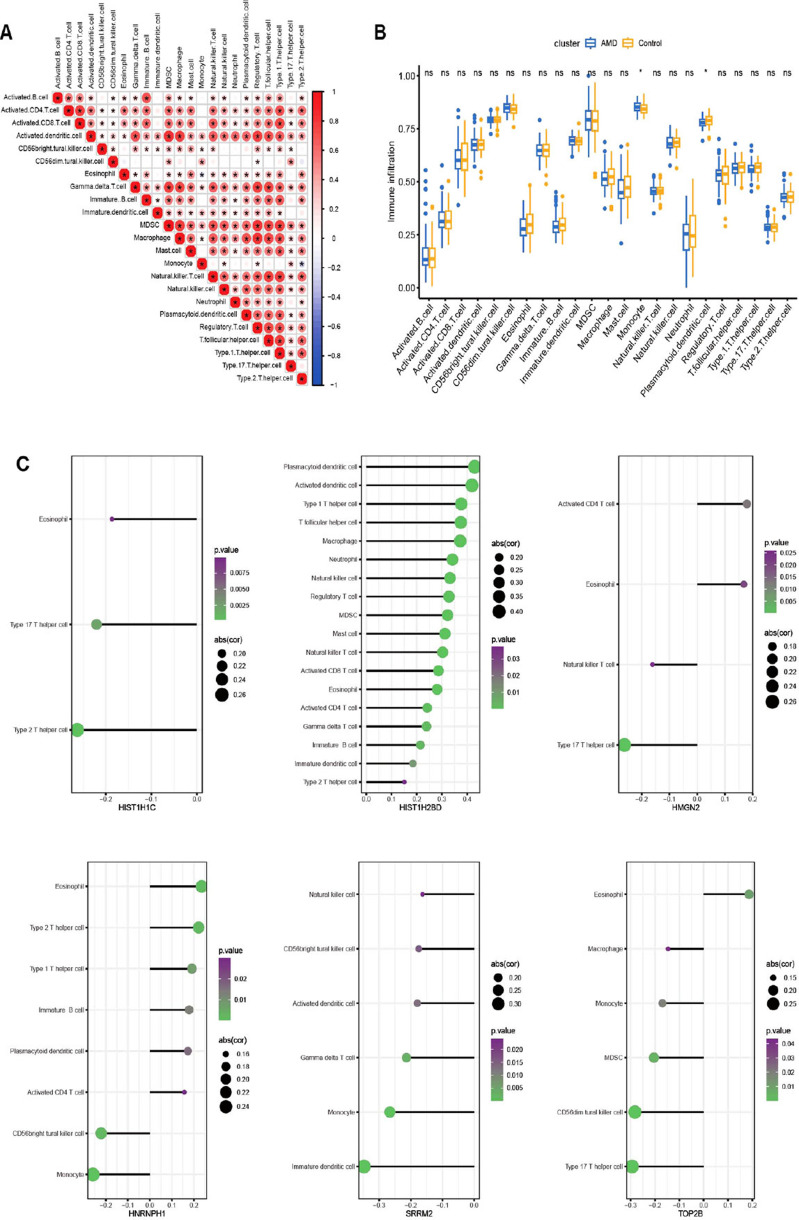
Evaluation of immune cell enrichment. (**A**) The enrichment correlation between immune cells. (**B**) Abundance of immune cells was compared between AMD and control groups. (**C**) The correlation between hub gene expression and immune cell enrichment. Only results with statistical significance (*p* < 0.05) were shown.

The immune cell enrichment was assessed with the ssGSEA algorithm using the “GSVA” R package based on the merged data matrix (Fig. **6A**). A comparison was made between AMD and control samples to determine any differences. The findings revealed that the infiltration levels of two out of the 23 immune cells, namely monocyte and plasmacytoid dendritic cells, exhibited differential infiltration (Fig. **6B**). Additionally, the correlation of the immune cell enrichment with six hub gene expressions was analyzed, with only results displaying a *p*-value < 0.05 being presented (Fig. **6C**).

### 3.6 Consensus Molecular Clustering based on Hub Genes

**Fig. (7):**
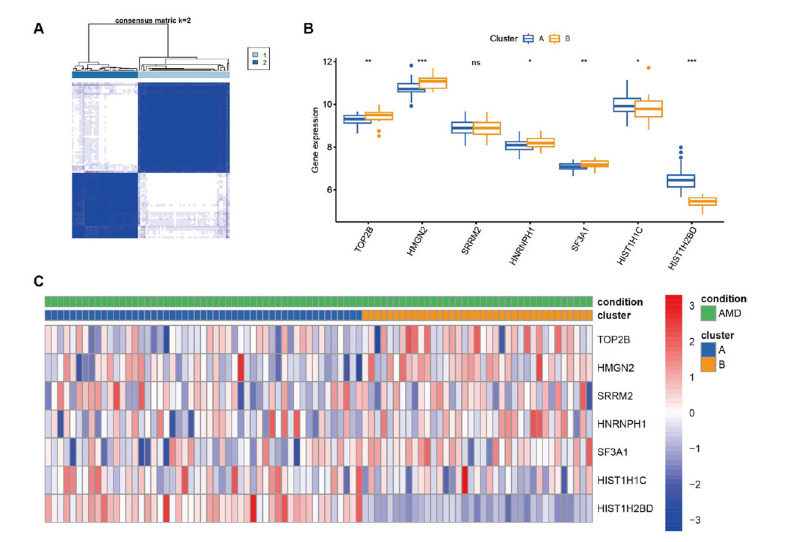
Consensus molecular clustering based on hub genes. (**A**) Consensus matrix heatmap illustrates the identification of several clusters and their correlation area. (**B**) Differential expression of hub genes between cluster (**A**) and (**B**). (**C**) Heatmap of hub genes.

**Fig. (8):**
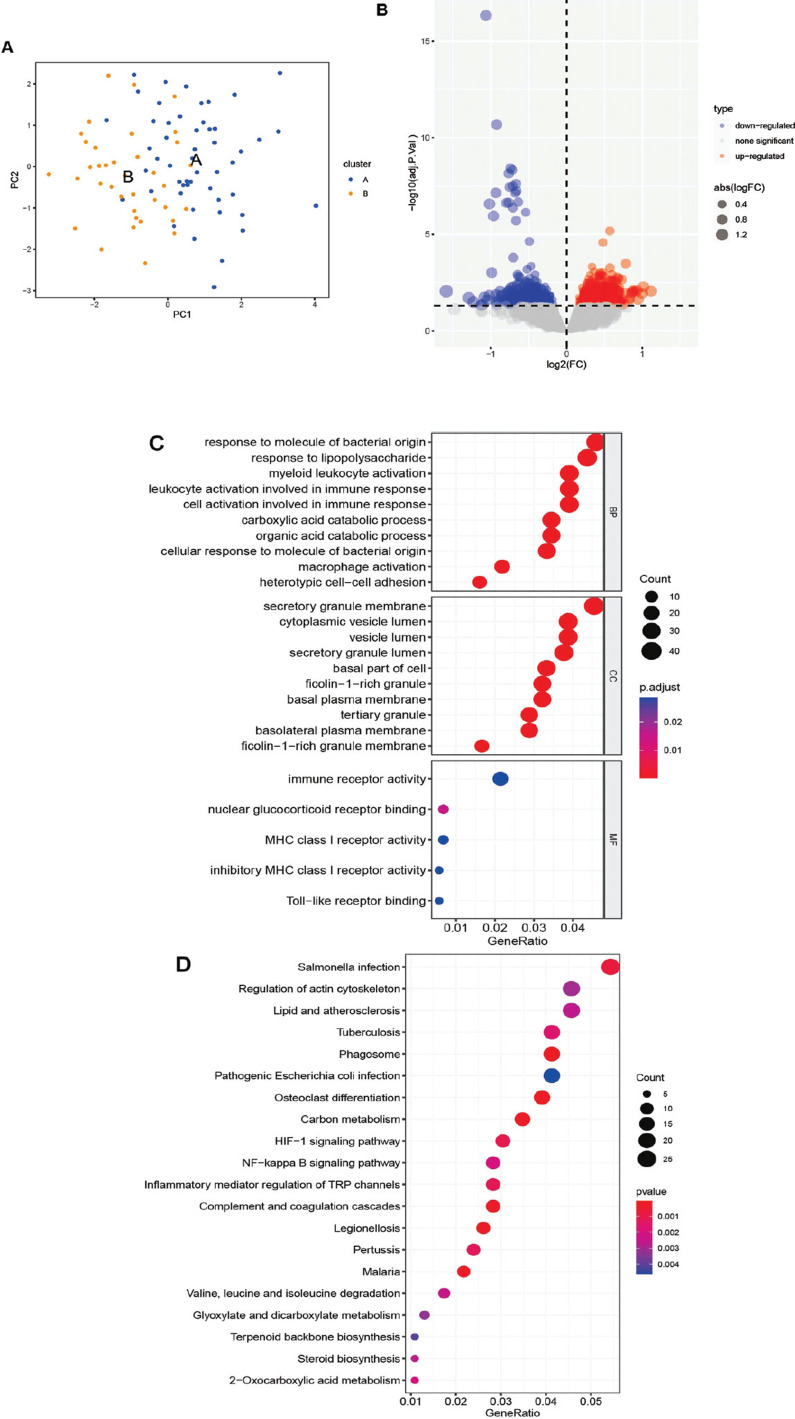
Difference between clusters A and B. (**A**) PCA plot of AMD patients in two clusters. (**B**) Volcano plots of DEGs between clusters A and B. (**C-D**) GO and KEGG enrichment analyses of DEGs between clusters (**A**) and (**B**). The top 20 were displayed.

In this research, seven hub genes in total were incorporated. A consensus clustering algorithm was employed to categorize patients into distinct clusters using the R package “ConsensusClusterPlus” and consider hub gene expression in AMD. Subsequently, two clusters were successfully identified (Fig. **7A**). The expression patterns and heatmap of these seven hub genes were visually presented (Fig. **7B** and **C**). Furthermore, a principal component analysis (PCA) plot was generated, clearly illustrating the discernible separation between the two clusters (Fig. **8A**). To clarify the functional differences between the various subtypes, a differential analysis of the two clusters was then conducted (Fig. **8B**), along with an enrichment analysis of DEGs. According to the analysis of GO, these DEGs were primarily associated with the response to molecules of bacterial origin in biological processes, the secretory granule membrane in cellular components, and the activity of immune receptors in molecular function (Fig. **8C**). The KEGG analysis demonstrated enrichment in salmonella infection, regulation of actin cytoskeleton, and lipid and atherosclerosis pathways (Fig. **8D**).

### 3.7 Features of Lactylation-related Key Genes

**Fig. (9):**
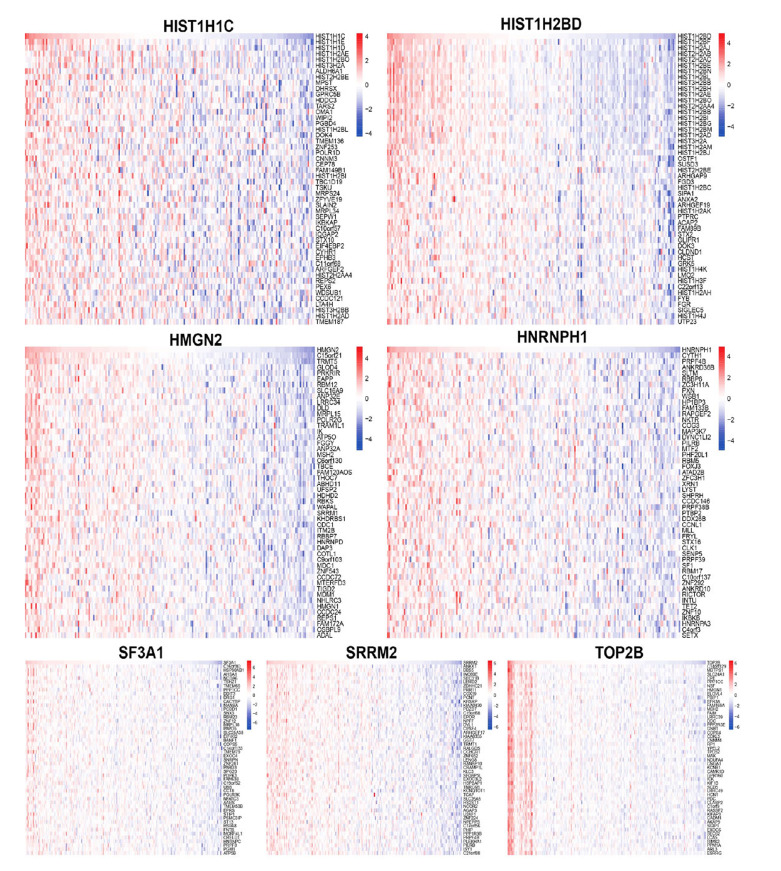
The co-expressed genes with hub genes in AMD samples. The heatmap showed top 50 co-expressed genes with seven hub genes in AMD samples.

**Fig. (10):**
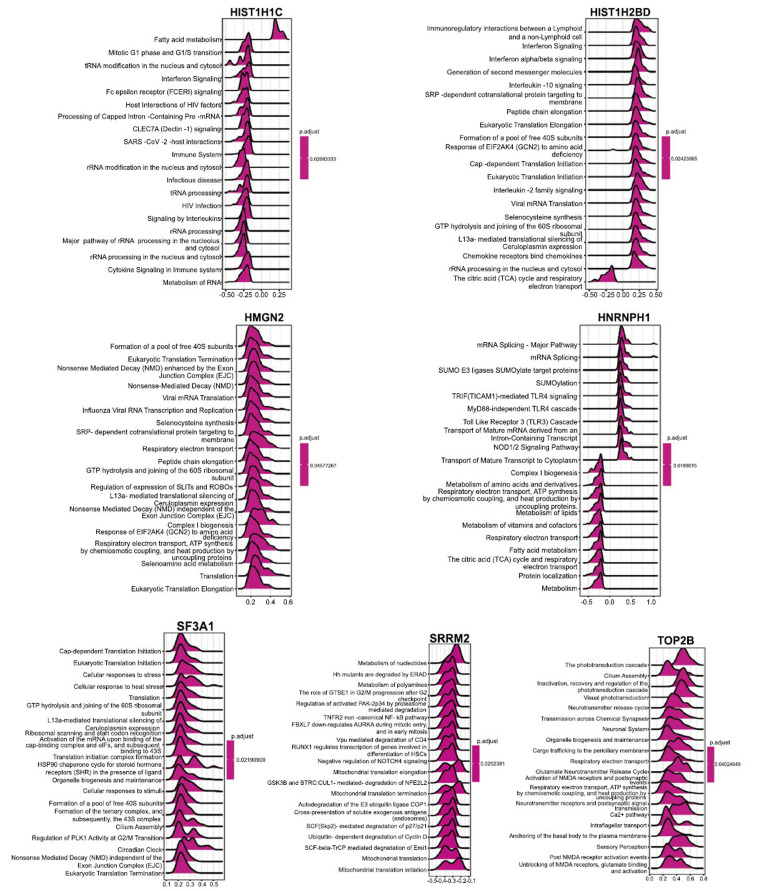
GSEA of key genes. The top 20 GSEA terms of seven hub genes were presented.

Furthermore, we analyzed the correlation between levels of seven key genes and the other genes in the expression matrix, and the heatmap displayed the top 50 genes with the strongest positive correlation (Fig. **9**). For instance, a positive correlation was observed between *HIST1H1C* and HIST1H1E, HIST1H1D, HIST1H2AE, and HIST1H2BO. Subsequently, a GSEA was conducted using Reactome to assess the signaling pathways associated with the 7 hub genes (Fig. **10**). The analysis revealed that *HIST1H1C* was linked to various pathways, including Fatty acid metabolism, Mitotic G1 phase and G1/S transition, Interferon Signaling, and Fc epsilon receptor signaling.

### 3.8 Establishment of the miRNA-TF-genes Network

**Fig. (11):**
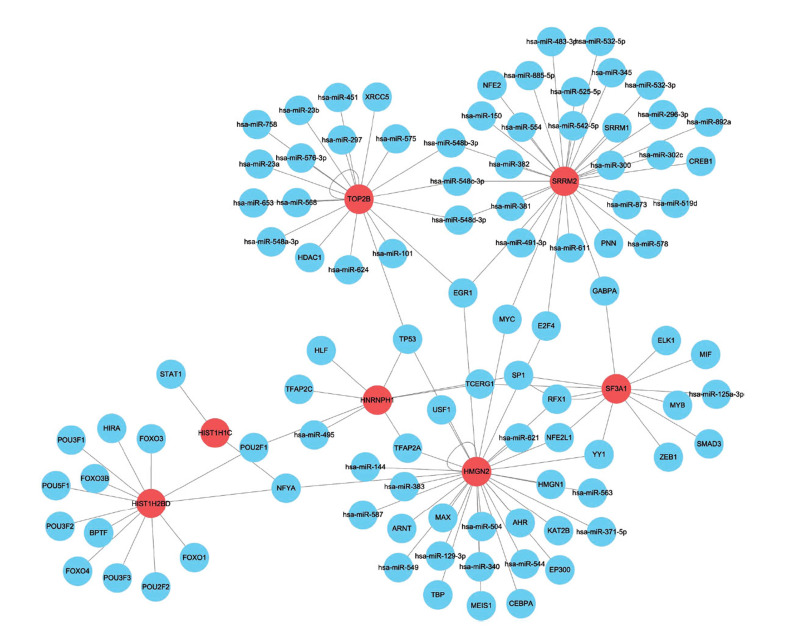
Construction of the miRNAs-TFs- hub genes networks. The miRNAs and TFs potentially regulating the 7 hub genes were searched on the RegNetwork database. The red color indicates the hub genes, and the blue color indicates the miRNAs or TFs. The lines represent predicted interactions.

MicroRNAs (miRNAs) and transcription factors (TFs) are key gene regulators involved in the regulation of cellular processes in AMD [21-24]. To elucidate the potential regulatory networks of the key genes, we explored the microRNAs and transcription factors potentially regulating these 7 key genes on the RegNetwork database, and the miRNA-TF- gene network was constructed by merging the regulatory pairs (miRNA-gene, TF-gene) (Fig. **11**). The network included 103 nodes (48 TFs, 48 miRNAs, 7 genes). For example, *HIST1H1C* was possibly modulated by transcription factors such as STAT1 and NFYA.

### 3.9 Validation of Expression of the Key Genes

The levels of seven key genes in the serum from AMD patients and healthy control individuals were measured using RT-qPCR. As shown in Fig. (**12**), all 7 hub genes, including *TOP2B, SRRM2*, *HNRNPH1, HMGN2, HIST1H1C, SF3A1,* and *HIST1H2BD* were down-regulated in AMD patients compared with control individuals, which suggested their potential value in AMD diagnosis.

## 4 DISCUSSION

AMD is a highly prevalent and progressive ocular ailment that predominantly affects the elderly demographic [10]. This condition presents a substantial public health issue as it has the capacity to induce profound visual impairment or complete blindness in older individuals [25, 26]. The macula, responsible for central vision and crucial visual activities such as reading, driving, and facial recognition, is primarily affected by this disorder. The etiology of AMD is intricate and multifaceted, encompassing a confluence of genetic, environmental, and lifestyle factors [27, 28].

Numerous research has been undertaken to elucidate the mechanisms and ascertain the risk factors associated with the occurrence and development of AMD. In recent years, substantial advancements have been achieved in comprehending the intricate molecular pathways implicated in AMD. Within these pathways, emerging evidence suggests that post-translational modifications (PTMs) are significantly involved in the development and progression of AMD [29-31]. PTMs are indispensable for regulating protein function and various cellular processes.

Lactylation, a PTM, has gained considerable prominence due to its involvement in the addition of lactate molecules to proteins. This modification has emerged as a pivotal regulatory mechanism in various biological processes, encompassing metabolism, signal transduction, and gene expression [32-34]. The versatility of lactylation offers a valuable avenue for the modification of proteins, peptides, carbohydrates, and other biomolecules, leading to alterations in their properties and activities. The introduction of lactyl groups has the potential to augment the stability, solubility, and bioavailability of these biomolecules, as well as impart desirable functionalities such as improved cell penetration or prolonged circulation time [35-37]. Furthermore, the process of lactylation has the potential to regulate the interactions between biomolecules and their respective target receptors, thereby exerting an influence on crucial biological processes including protein-protein interactions, enzyme activity, and immune responses [38]. However, the specific mechanisms through which lactylation modifications contribute to the progression of AMD are still not comprehensively understood.

In this study, we employed an integrated bioinformatics technique for the identification of hub genes related to AMD. Initially, we conducted a screening of DEGs between the AMD and control groups. Subsequently, GO and KEGG analyses were applied to elucidate the functional characteristics of the DEGs. The results of this research indicated the relation of DEGs with cell growth, response to radiation, and alcohol metabolic processes in terms of BP. Furthermore, the CC analysis indicated the association of DEGs with the collagen-containing ECM, apical part of the cell, and cell projection membrane. Lastly, the MF analysis highlighted the involvement of DEGs in terms of DNA-binding transcription repression activity and RNA polymerase II-specific activity. The analysis of KEGG results revealed enrichment in pathways such as lipid and atherosclerosis, axon guidance, Wnt signaling pathway, carbon metabolism, morphine addiction, and TGF-beta signaling pathway.

Next, we acquired lactylation-related genes and specifically chose these from the pool of DEGs. For high-dimensional data such as array datasets, the traditional multifactor logistic regression model is not suitable because the gene multicollinearity may lead to overfitting of the model. Thus, we employed three machine learning algorithms commonly used for the selection of feature genes [39, 40] to identify feature genes. LASSO regression is a machine learning technique identifying the λ value under the least classification error [18]. In this study, LASSO analysis identified 36 characteristic genes. Random forest screened ten important genes. SVM-RFE is a surveillant machine learning method exploring the beast variables by subtracting the feature vector generated by the SVM [20]. As for the SVM-RFE algorithm, the classifier achieved the minimum error when the feature count reached 68. Upon intersecting the results, 7 common genes (*SRRM2*, *HMGN2*, *TOP2B*, *SF3A1*, *HIST1H1C*, *HNRNPH1*, and *HIST1H2BD*) were regarded as key genes in AMD. Moreover, RT-qPCR analysis revealed the down-regulation of these 7 genes in AMD patients relative to healthy controls.

MiRNAs and transcription factors are critical regulators of gene expression at the posttranscriptional and transcriptional levels. The miRNAs-TFs-genes networks are suggested to be useful in exploring the pathobiological processes and molecular basis in diseases [41-43]. In order to understand the molecular mechanisms of seven key genes, we investigated the miRNAs and TFs associated with these seven target genes. Total 48 TFs, and 48 miRNAs were included in this network. For instance, the hub gene *HIST1H1C* was predicted to be regulated by TFs such as STAT1 and NFYA, and *HIST1H1C* has been reported to aggravate diabetic retinopathy by promoting inflammation, autophagy, glial activation and neuron loss [44], while STAT1 has been reported to be involved in AMD progression [45]; the hub gene *TOP2B* was predicted to be regulated by miR-568, miR-101, HDAC1 and others. HDAC1 has been revealed as a hub gene related to proliferation or differentiation in AMD development [46]. Exploration and construction of the regulatory networks of these hub genes may help understand the molecular basis of AMD [47].

## CONCLUSION

Our investigation has identified seven lactylation-related hub genes in AMD, namely *SRRM2*, *HMGN2*, *TOP2B*, *SF3A1*, *HIST1H1C*, *HNRNPH1*, and *HIST1H2BD*. The potential biological functions and signaling pathways of these hub genes were explored using enrichment analysis. Immune infiltration analyses also suggested that the hub genes were linked with the immune state in AMD. The miRNA-TF-genes networks were established to further explore the underlying mechanism of these hub genes. Future studies are needed to validate the potential interactions deduced in these networks experimentally. The findings of our study might offer a novel avenue for exploring the pathophysiology of AMD.

## Figures and Tables

**Fig. (12):**
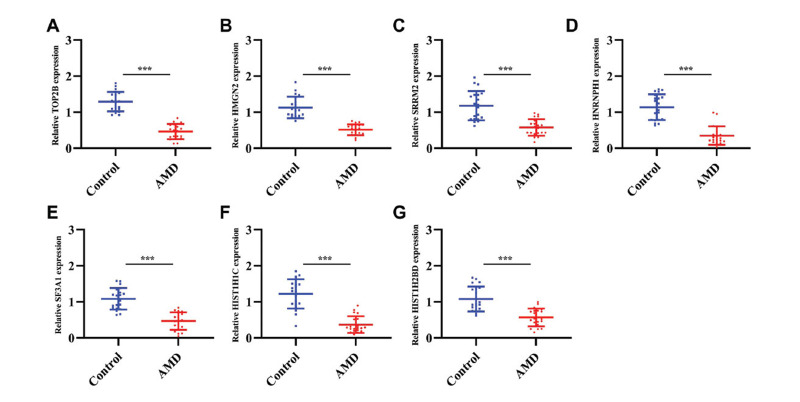
Expression validation of seven key genes. RT-qPCR validated the down-regulation of (**A**) *TOP2B,* (**B**) *HMGN2,* (**C**) *SRRM2*, (**D**) *HNRNPH1,* (**E**) *SF3A1,* (**F**) *HIST1H1C* and (**G**) *HIST1H2BD* in the serum of AMD patients compared with control individuals.

## Data Availability

The datasets generated and/or analyzed during the current study are available in the GEO repository https://www.ncbi.nlm.nih.gov/geo/query/acc.cgi?acc=GSE 29801 and https://www.ncbi.nlm.nih.gov/geo/query/acc.cgi? acc=GSE50195.

## LIST OF ABBREVIATIONS

AMD: Age-related Macular Degeneration
BP: Biological Processes
CC: Cellular Components
DEGs: Differentially Expressed Genes
GEO: Gene Expression Omnibus
GO: Gene Ontology
GSEA: Gene Set Enrichment Analysis
KEGG: Kyoto Encyclopedia of Genes and Genomes
Lasso: Least Absolute Shrinkage and Selection Operator
MF: Molecular Functions
MiRNAs: microRNAs
PCA: Principal Component Analysis
PTM: Post-translational Modification
SVM: Support Vector Machines
TFs: Transcription Factors
